# Managing broiler production challenges at high altitude

**DOI:** 10.1002/vms3.784

**Published:** 2022-03-15

**Authors:** Fariborz Khajali

**Affiliations:** ^1^ Department of Animal Science Faculty of Agriculture Shahrekord University Shahrekord Iran

**Keywords:** altitude, chicken, feed, hypoxia, metabolic disorders

## Abstract

This review covers the challenges of broiler chickens at high altitude, with the focus on growth performance and physiological response. The review also sheds light on nutritional and management interventions that help overcome the challenges raised at high altitude. Reduced concentration of atmospheric oxygen is by far the biggest challenge that remarkably affect growth performance and livability of broiler chickens reared in high altitude area. Broiler chickens have endured intensive genetic selection, which potentially predispose them to several metabolic disorders. Hypoxia is an overriding factor that may increase the incidence of metabolic disorders, mainly ascites syndrome at high altitude. Commercial broiler strains cannot fully achieve their genetic potential when raising at highland regions. Careful nutrition and management considerations are required to prevent metabolic disorders when raising broilers at high altitude. In ovo or in‐feed nutraceuticals such as l‐carnitine and guanidinoacetic acid as well as pharmaceuticals, texture of feed and the use of proper sources and levels of dietary energy and protein are important factors that need to be carefully considered for rearing broiler chickens at high altitude. Management strategies such as lighting programs have been shown to be effective to circumvent ascites prevalence. Special breeding programs may also be considered to develop strains with resistance to ascites.

## Graphical abstract

High altitude (i.e hypobaric hypoxia) is associated with the proliferation of red blood cells (i.e increased hematocrit). Accelerated production of red blood cells leads to a higher number of immature cells that in one hand are impotent in carrying oxygen and in the other hand increases blood viscosity. Consequently, the right side of the heart is challenged with pumping more viscous blood to the lungs. The pressure overload eventually develops into right ventricular hypertrophy and pulmonary arterial hypertension syndrome.

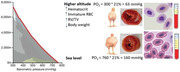

## 1 INTRODUCTION

High‐altitude environments impose several challenges to broiler chickens, including hypobaric hypoxia, dehydration and cold (Parr et al., 2019). Although the percentage of oxygen in air is constant at different altitudes, the drop in the barometric pressure of the atmosphere at higher altitude reduces the partial pressure of oxygen. Hence, oxygen availability is reduced in high altitude area. The driving force for gas‐exchange at the blood–air barriers (alveolar–capillary junctions) are partial pressures of O_2_ and CO_2_ and the gradient of O_2_ and CO_2_ in the air and blood capillaries. At high altitude, a drop in the partial pressure of O_2_ in air capillaries will impact gas exchange. As a result, oxygen gradient against the partial pressure of O_2_ in deoxygenated blood is reduced, leading to reduced diffusion of O_2_ into the arterial blood. Concurrently, the opposite scenario happens for venous CO_2_ with the result of retaining a higher concentration of CO_2_ in the arterial blood. The ramification of these occurrences is hypoxemia, which causes several physiological adjustments. The most prominent response to hypoxemia is transcriptional changes in hypoxia‐inducible factors, mainly HIF‐1α (Semenza, 2012). Research has shown that HIF‐1α is associated with the development of ascites syndrome in broiler chickens (Zhang et al., 2013).

It is worth noting that broiler chickens (*Gallus gallus*) have endured intensive genetic selection for rapid growth rate for decades. This has resulted in disproportionate growth of oxygen‐supplying organs (i.e., heart and lungs) and oxygen‐requiring tissues (muscles), rendering broilers in a mild hypoxemic condition (Decuypere et al., 2000; Wideman et al., 2013). Raising rapid‐growing broiler chickens with pre‐existing hypoxemia at high altitude intensifies hypoxemia and put their lives at stake. Therefore, proper nutritional interventions and management approaches are needed to control metabolic diseases, particularly ascites syndrome, in broilers reared at high altitudes.

## 2 HIGH ALTITUDE AND PHYSIOLOGICAL CONSEQUENCES

There is a number of cell‐signalling mechanisms by which cells respond to hypoxia. The discovery of cellular mechanisms to sense and adapt to different oxygen concentrations has been the focus of intensive research and the pioneers who established the basis as to how oxygen levels affect physiological function of cells were awarded the 2019 Nobel Prize in Physiology or Medicine (https://www.nobelprize.org/prizes/medicine/2019/summary). Hypoxia disturbs the redox state of electron transport proteins of the proximal complexes upstream from the oxidase. Mitochondria and in particular complex III generate oxidants which stabilize the hypoxia‐inducible factor ‐1 alpha (HIF‐1α) during hypoxia (Gaur et al., 2021). HIFs are transcriptional activators that function as dominant regulators of oxygen homeostasis in animals and birds (Semenza, 2012).

Hypoxia‐inducible factor 1 (HIF‐1) that is expressed by all cells consists of two subunits: HIF‐1α and HIF‐1β. In normoxic conditions, a specific proline residue in HIF‐1α undergoes hydroxylation, which results in binding with another protein by virtue of oxygen as a substrate. However, this biochemical process is inhibited under hypoxic conditions, leading to up‐regulation of HIF‐1α (Semenza, 2012). The expression of a set of hundreds of genes is increased in response to hypoxia in an HIF‐1‐dependent manner. Meanwhile, the expression of another set of hundreds of genes is decreased in hypoxia in an HIF‐1‐dependent manner (Wang et al. 2013; Semenza, 2012). These transcriptional changes dysregulate many physiological processes including erythropoiesis, pulmonary arterial function and cell division (Malila et al., 2019).

It is well established that increased erythropoiesis in response to hypoxia leads to physiologic polycythaemia (increased production of red blood cells). Polycythaemia increases the haematocrit value and enhances the oxygen‐carrying capacity of blood (Wideman et al., 2013). Polycythaemia is associated with increased production of immature red blood cells, which are not competent in carrying oxygen due to reduced flexibility and impaired deformability (Luger et al., 2003). This phenomenon along with increased haematocrit increase the blood's viscosity and thereby increase the resistance to blood flow (Wideman et al., 2013). The pulmonary arterial response of broilers to hypoxia and hypoxemia is vasoconstriction and hypertension (Martinez‐Lemus et al., 2003; Wideman et al., 2013). Increased resistance to blood flow and pulmonary arterial hypertension faces the right ventricle of heart with a pressure overload, which eventually develop into right ventricular hypertrophy and pulmonary arterial hypertension syndrome (also called ascites syndrome).

**FIGURE 1:**
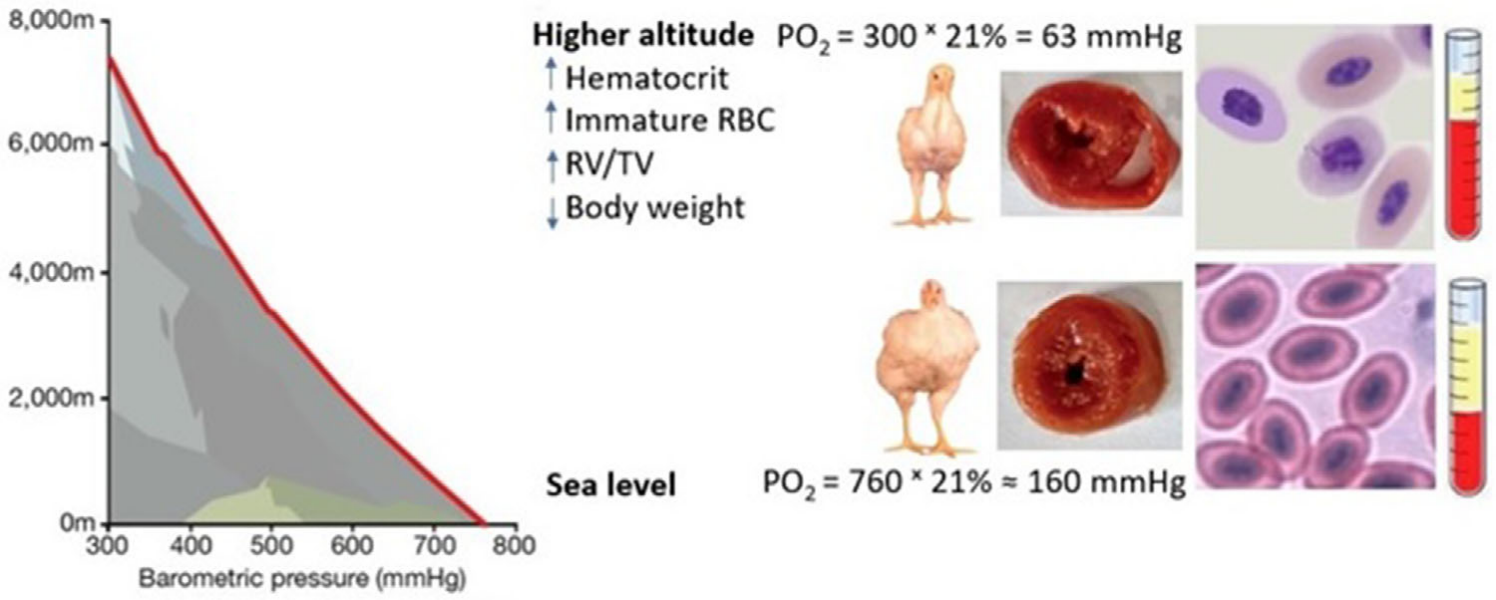
Physiological responses of broiler chickens to high altitude

Other important physiological responses to high altitude hypoxia include amplified generation of reactive oxygen species (ROS; Gaur et al., 2021), downregulation of cardiac β‐adrenergic receptors (Hassanzadeh et al., 2001), increased release of catecholamines (Rostrup, 1998) and switching metabolism from oxidative phosphorylation to glycolytic oxidation (Tirpe et al., 2019). Figure 1 depicts the main physiological changes in broiler chickens raised at high altitude.

## 3 ASCITES SYNDROME: THE MAIN RAPID‐GROWTH PROBLEM AT HIGH ALTITUDE

Broiler chickens have endured intensive genetic selection for rapid growth and higher breast mass, leading to strikingly different characteristics to their ancestors. Extraordinary growth rate of a broiler chicken turns a 40 g broiler chick at hatch into a 4000 g chicken at 56 days of age (Khajali & Wideman, 2016). Improved performance of broiler chickens has been evolved by virtue of genetics and breeding programs. A report that compared genotypes representative of broiler chickens of the years 1957 and 2005 indicated that body weight was improved by 460% (0.90 vs. 4.20 kg) and feed conversion ratio (FCR) improved by 50% (2.854 vs. 1.918; g of feed/g of BW gain) over a 56‐day period from 1957 to 2005 (Tavarez & Solis de los Santos, 2016). The report also indicated that broiler breeding companies have placed intensive selection pressure on breast meat yield over the past 20 years. Consequently, the growth rate of skeletal muscles outpaced the growth rate of the heart and lungs. Therefore, a mismatch has been evolved between oxygen‐demanding organs (i.e., muscles) and oxygen‐supplying organs (i.e., heart and lungs), which triggers hypoxemia and potentially predispose broilers to ascites syndrome (Decuypere et al., 2000).

Arce‐Menocal et al. (2009) compared two strains of broilers and found that the strain with greater body weight gain also had greater ascites‐related mortality, which accounted for nearly 50% of the total mortality when reared to 42 day of age.

## 4 HIGH ALTITUDE AND BROILER PERFORMANCE

Using respiratory chambers, Beker et al. (2003) evaluated the effects of a wide range of atmospheric oxygen concentration (12, 14, 16, 18 and 20.6%) on growth performance and the development of ascites syndrome in broiler chickens. They found that reductions in atmospheric oxygen concentration significantly reduced growth performance and exacerbated ascites criteria. Wight gain of broiler chickens subjected to 12%, 14%, 16%, 18% and 20.6% oxygen concentration was 138, 287, 353, 356 and 371 g at 14 days of age, respectively. It is evident that broilers reared under atmospheric oxygen concentrations of 12, 14, 16 and 18% had weight losses of 62.8%, 22.6%, 4.9% and 4.0%, respectively, when compared to chickens raised at sea level. Haematocrit values of 49, 42, 36, 33 and 32 and ascites scores of 3.00, 2,23, 0,67, 0.38 and 0.25 were reported for broiler chickens raised at 12, 14, 16, 18 and 20.6% atmospheric oxygen concentration. Some studies, therefore, evaluated the response of different strains of broiler chickens under high altitude to identify appropriate strains of broilers.

Kalia et al. (2017) conducted an experiment to identify the suitable broiler strain for a high‐altitude region (3500 m). One‐week old chicks from three broiler strains (Vencobb, RIR cross‐bred and Hubbard) were randomly selected and distributed equally into three groups. Live performance of all the chicks were recorded on the same experimental diet. RIR cross‐bred had the highest body weight gain and the best FCR compared to other strains. Body weight gain and FCR for RIR cross‐bred, Vencobb and Hubbard were 288, 254 and 250 g/b and 3.36, 3.94 and 4.36, respectively. It is worth noting that the objective weigh gain and FCR suggested by Hubbard manual for broilers for the same period (7–35 days) is 2058 g/b and 1.48, respectively. It is evident that Hubbard broilers have only gained 12% of their genetic potential when subjected to a high altitude of 3500 m.

## 5 HIGH ALTITUDE AND IMMUNITY

Hypoxia impacts the differentiation and functions of immune cells including T and B lymphocytes (McNamee et al., 2013) and as a consequence influences the levels of immunoglobulins and cytokines. The levels of cytokines can also be influenced by hypoxia, thereby disrupting the balance between proinflammatory and anti‐inflammatory cytokines. Moreover, Wang et al. (2019) found that the levels of IgA, IgG and IL‐10 were significantly higher in birds reared at high altitude.

## 6 NUTRITIONAL STRATEGIES AT HIGH ALTITUDE

### 6.1 In ovo feeding

The concept of in ovo feeding is providing essential nutrients to developing embryos or newly hatched chicks until they obtain a fully functional digestive system. Saki et al. (2013) studied the effect of in ovo feeding of L‐arginine (ARG) in the prevention of ascites syndrome in broiler chickens reared at high altitude. On day 5 of incubation, one group was injected with 0.5 ml of ARG (20 mg/ml) and another group remained untreated and served as control.  Their finding indicated that in ovo injection of ARG significantly decreased pulmonary hypertension syndrome mortality of broilers (18.8% vs. 43.8%). In another study, in ovo injection of thyroxine was associated with improved chick quality and reduced incidence of ascites syndrome in broiler chickens (Afsarian et al., 2018). In ovo injection of nutrients in the embryonated eggs may exert beneficial effects through changes in gene regulation of newly hatched broiler chicks and needs further investigation (Jha et al., 2019).

### 6.2 Feed restriction

Feed restriction was first used as a means to control ascites syndrome at high altitude (Arce et al., 1992). The incidence of ascites syndrome was significantly reduced by the application of feed restriction at early age of broilers (Acar et al., 1995; Arce et al., 1992). Feed restriction by limiting access to feed for just 8 h a day during early life was also shown to be effective to control ascites mortality of broilers (Camacho et al., 2004). Khajali et al. (2007) reported a reduction in mortality from ascites by imposing a skip‐a‐day feed removal at early age of broiler chickens. Feed restriction has been sometimes applied by limiting the quantity of feed to 60% of the free access condition with the result of reduced ascites mortality (Mohammadalipour et al., 2017). In practice, however, early feed restriction methods are not recommended because broiler chicks have a poorly mineralized skeleton at hatch and the first 2 weeks post‐hatch is the phase of rapid mineralization of skeleton (Angel, 2007; Rath et al., 2000). Therefore, feed restriction during this time will dramatically impede physiological growth and development of skeleton and may result in a number of skeletal problems.

### 6.3 Feed texture

Feeding pelleted feed compared to crumbles increased ascites‐related mortality in broiler chickens (Arce‐Menocal et al., 2009). Feeding a mash or small particle crumbled starter diet to a broiler of 18–20 days of age is recommended by broiler chicken nutrition guidelines (Hubbard, 2005). It is also suggested to provide a mash grower feed when raising broilers at high altitude. Mash feed slows down the intake of feed and, thus, helps slow down growth rate and reduces the development of ascites syndrome (Urdaneta‐Rincon & Leeson, 2002). Robertson (2005) reported that feeding crumbles to turkey poults increased round heart disease incidence starting at 6 days of age.

### 6.4 Energy source

It is well established that feeding diets with condensed energy to broilers is associated with the development of ascites syndrome (Camacho‐Fernandez et al., 2002). The reason is high energy increases the metabolic rate, albeit with increased ROS production. The type of energy substrates is equally important as the energy content. Fat supplementation of broiler chickens was reported to increase the incidence of ascites at high altitude (Khajali & Fahimi, 2010). It is presumed that fats increase the birds’ demand for oxygen. Increased oxygen demand exacerbates hypoxic conditions under high altitude and leads to ascites syndrome. The type of fatty acids has also a vital role in the development of ascites. Feeding oils with high proportion of n‐3 fatty acids alleviated broiler ascites when compared to the oil source with high proportion of n‐6 fatty acids (Rostami et al., 2016). It is suggested that incorporation of n‐3 fatty acids in membranes of red blood cells increases the fluidity of the membranes and the deformability of red blood cells (Walton et al., 2001).

### 6.5 Protein source

It has been established that feeding reduced‐protein diets to broiler chickens is associated with the development of ascites syndrome (Behrooj et al., 2012; Sharifi et al., 2015a, 2016). Reduced production of uric acid, reduced intake of arginine and intensive lipogenesis are the factors involved in the development of ascites syndrome when reduced‐protein diets are fed (Khajali & Wideman, 2016). Protein quality is also a crucial factor in the development of ascites syndrome. Substitution of soybean meal with canola meal was reported to be linked with the incidence of ascites in broiler chickens (Izadinia et al., 2010). The higher incidence of ascites was thought to be related to reduced intake of arginine. Canola meal is also a poor source of genistein compared to soybean meal. Genistein (5,7‐dihyroxy‐3‐(‐4‐hydroxyphenyl)chromen‐4‐one) is an isoflavone abundant in soybean that exerts beneficial effects on the vasculature and helps to combat pulmonary arterial hypertension (Sánchez‐Gloria et al., 2020).

### 6.6 Vitamins and microelements

Potential role of ROS in the etiology of ascites syndrome has been well documented. Hence, supplementation of broiler diets with antioxidant vitamins and microelements has been evaluated as an approach to control broiler ascites. Hassanzadeh et al. (1997) reported that vitamin C supplementation at 500 mg/kg significantly decreased ascites mortality in broiler chickens. Khajali and Sharifi (2018) were able to prevent ascites syndrome in broiler chickens fed a reduced‐protein diet at high altitude. Ruiz‐Feria (2009) supplemented broiler diets with vitamins C and E and observed reduced pulmonary arterial pressure and reduced ascites index. Adding vitamin E or C alone has not always be associated with reduced ascites mortality in broilers (Khajali & Fahimi, 2010; Villar‐Patino et al., 2002). The lack of improved response to single antioxidants was confirmed by several researchers (Bottje et al., 1997; Villar‐Patino et al., 2002). This finding suggests that the antioxidant network is a complex nexus of several antioxidants working in concert, and it is unlikely that one single antioxidant protect well against ROS in the absence of other elements.

Effects of selenium as an antioxidant microelement on the incidence of ascites syndrome in broilers have been studied. Ozkan et al. (2007) reported a significant improvement in ascites‐related variables of broiler chickens fed with different sources of selenium ranging from 0.15 to 0·30 mg/kg. Moghaddam et al. (2017) evaluated the effects of different sources of selenium (organic and nano) on broiler ascites criteria. They found that nano‐selenium at 0.30 mg/kg could prevent right ventricular hypertrophy in broiler chickens.

### 6.7 Nutraceuticals

The term “nutraceutical” is referred to a substance that is considered a food or part of a food, which confers medical or health benefits and prevents disease. CoQ_10_ at 40 mg/kg was reported to have a beneficial effect in reducing ascites mortality in broiler chickens (Geng et al., 2004). Prophylactic effect of CoQ_10_ on preventing ascites was confirmed in several studies (Faraji et al., 2019; Sharifi et al., 2016). CoQ_10_ exerts its beneficial effects on the development of ascites syndrome through strong antioxidant role.

l‐carnitine (LC) has been used to improve cardiopulmonary function and avoid ascites in broiler chickens (Sharifi et al., 2015b). Yousefi et al. (2013) evaluated different levels of LC (50, 100 and 150 mg/kg) on right ventricular hypertrophy in broilers and found 100 mg/kg as the optimal dose. LC improves broiler hypertensive response through several ways: First, as an antioxidant factor, second, as an antihyperlimidemic agent, third, by increasing nitric oxide through reduction in the activity of arginase and elevation in the activity of nitric oxide synthase. Fourth, by transcriptional changes in metabolic and antioxidant genes (Wang et al., 2013; Yousefi et al., 2013).

Guanidinoacetic acid (GAA) is a heat‐stable nutraceutical that could spare arginine (ARG) requirements of broiler chickens (Khajali et al., 2020). Arginine is the substrate from which NO is synthesized. NO is a potent vasodilator that prevents pulmonary hypertension. A crucial role for ARG and NO in preventing ascites syndrome in broilers has been documented (Khajali & Wideman, 2010). In a recent study, the influence of different levels of GAA on right ventricular hypertrophy in broiler was studied (Ahmadipour et al., 2018). The finding indicated that GAA caused a significant increase in plasma level of NO with concomitant decrease in ascites‐related variables.

### 6.8 Pharmaceuticals

Furosemide is a carboxylated sulfonamide used as a diuretic to inhibit electrolyte reabsorption in the ascending part of Henle's loop (Wideman et al., 1995). By preventing sodium and fluid retention, furosemide relieves edema and ascites in human patients with congestive heart failure or hepatic cirrhosis (Laffi et al. 1993). High amount of sodium supplied in the diet or drinking water results in the development of ascites in broilers (Julian et al., 1992; Wideman, 1988). Consequently, diuretic therapy to reduce sodium and fluid retention might be expected to reduce ascites mortality. Furosemide also acts as a pulmonary vasodilator in broilers (Wideman et al., 1995).

Prophylactic use of metaproterenol, a bronchodilator drug, has been examined as a means to prevent ascites in broilers. Vanhooser et al. (1995) administered a dose of 2 mg/L of metaproterenol via drinking water to broilers reared at sea level (20.6% oxygen concentration) and high altitude (17.6% oxygen concentration). They found that metaproterenol completely prevented ascites at sea level and significantly reduced ascites incidence at high altitude.

Aspirin is a medication to relieve inflammation and fever as well as to avoid blood clots. Aspirin suppresses the production of inflammatory prostaglandins and thromboxanes by inactivation of the cyclooxygenase enzyme. Because ascites syndrome in broilers is associated with vasoconstriction and blood clotting, aspirin has been used to ameliorate the ascites condition. Balog et al. (2000) used aspirin at 0.05%, 0.1% and 0.2% in broiler diets and observed a reduction in ascites incidence at 0.20% aspirin compared with control (34% vs. 56%). Similar observation was reported by other researchers (Fathi & Haydari, 2016).

Probiotics have recently provided a potential pharmaceutical approach to counteract ascites mortality in broilers grown at high altitude. Kalia et al. (2017) used a probiotic (mainly composed of *Bacillus coagulans* and *Saccharomyces cerevisiae*) in broiler diets raised at high altitude. Their finding indicated that mortality from ascites was reduced in probiotic treated groups. Similar results have been previously reported (Saffar & Khajali, 2010). In a recent study, supplementation of a probiotic composed of *Lactobacillus plantarum* significantly decreased the mortality of chickens reared at high altitude (Wang et al., 2019). The possible mechanisms of action for probiotics to prevent ascites are (a) modulating bird's immune system, which could be induced and regulated by HIFs (McNamee et al., 2013), and (b) manipulating gut microbiota in a way less ammonia is produced (Chiang & Hsieh, 1995). The lower the production of intestinal ammonia, the lower the oxygen demand and lower the incidence of ascites. Research has shown that supplementing broiler diets with urease inhibitor decreased right ventricular hypertrophy and ascites (Anthony et al., 1994; Balog et al., 1994).

### 6.9 Phytochemicals

Phytochemicals are naturally occurring compounds in feedstuffs that can exhibit a potential for modulating metabolic disorders such as ascites syndrome. Wild celery (*Kelussia odoratissima*) is a rich source of bioactive compounds including phthalides, mainly z‐ligustilide, sesquiterpenes, flavonoids and polyphenols. Wild celery has shown to strongly suppress pulmonary hypertension and ascites in broiler chickens raised at high altitude (Ahmadipour et al., 2015). Research has proved z‐ligustilide as a significant substrate in relaxation of arterial smooth muscle cells (Kuang et al., 2008) and effective in inhibiting vascular remodelling (Lu et al., 2006). Monoterpenes (mainly carvacrol) were reported to attenuate pulmonary hypotension in broilers. Dietary inclusion of nettle (*Urtica dioica*), with high amount of carvacrol, reduced the incidence of ascites mortality in broiler chickens reared at high altitude (Ahmadipour & Khajali, 2019). Ethanol extract of elecampane (*Inula helenium* L.) rhizome (rich source of sesquiterpenes) has shown to attenuate ascites syndrome in broiler chickens (Abolfathi et al., 2021). Root extract of *Prosopis farcta* (rich source of flavonoids) has shown the potential to reduce the occurrence of ascites in broiler chickens (Shirzadi et al., 2020). Supplementing broiler diets with 3 g/ kg purslane (*Portulaca oleracea* L.) powder improved antioxidant status and reduced ascites incidence without affecting growth performance (Habibian et al., 2017). Purslane is a rich source of phenolic alkaloids (oleraceins) and α‐linolenic acid.

Pomegranate is a rich source of flavonoids, with the peel containing the highest flavonoid content and greatest antioxidant capacity compared to the seed and juice (Rafiei and Khajali, 2021). Feeding broiler chickens with pomegranate peel at 7.5 and 10 g/kg effectively subsided right ventricular hypertrophy and decreased ascites mortality (Ahmadipour et al., 2021). Inclusion of 5 g/kg garlic bulb in diets effectively reduced ascites incidence in broiler chickens (Varmaghany et al., 2015). Garlic composed of allicin, a sulphur‐containing compound, that exerts hypotensive effect.

## 7 MANAGEMENT STRATEGIES AT HIGH ALTITUDE

### 7.1 Eggshell temperature

Eggshell temperature (EST) during incubation has been reported to be associated with post‐hatch ascites incidence in broilers. Afsarian et al. (2016) periodically exposed hatching eggs for 1 h to an ambient temperature of 15°C, resulting in periodically lower EST of 25.5, 27.0, 32.5 and 36.0°C, respectively, at days 11, 13, 15 and 17 of incubation for a maximum period of 1 h. Periodically cooling of hatching eggs below 37.8°C EST reduced post‐hatch ascites mortality in chicks exposed to low environmental temperatures.

There are several factors that impact broiler productivity and livability at high altitude. A key factor that comes into play at high altitude is dehydration and excessive water/weight loss of broilers. Broiler chicks hatched at relative humidity of 52%–55% may be transported to high altitudes with relative humidity as low as 10%. In such circumstance, broiler chicks are rapidly dehydrated and died. Therefore, broiler houses should be equipped with foggers to provide necessary humidity (minimum 50%) before chick placement. Administration of betaine in feed or water may also be recommended due to its osmoregulatory effects (Kidd et al., 1997). Nevertheless, beneficial effects of betaine in alleviating broiler dehydration could be the subject of future research.

### 7.2 Brooding temperature

The brooding temperature during 1‐week post‐hatch has also been shown to have a significant influence on broiler liveability at high altitude. To avoid ascites, exposure to any periods of cold stress during the brooding period must be prevented because cold periods increase the metabolic rate (oxygen demand) and predispose birds to ascites later in life. A research study was carried out at 2600 m where the 35°C first week brooding temperature was compared with 32°. The temperature after 7 days was kept the same for both groups. The 32°C group of broilers had a total mortality of 9% (4%–5% from ascites) up to 38 days of age compared to only 3% total mortality for the 35°C group (Hubbard, 2005). Aviagen recommends that floor temperatures should be 28–30°C, air temperature should be 30°C and relative humidity between 60%–70% at placement.

### 7.3 Dehydration

Broiler chicks are hatched at a relative humidity between 52% and 54%. The relative humidity at high altitude hardly exceeds 20% in the summer and 40% in the winter. As soon as chicks are exposed to such a low humidity level, they become dehydrated. Humidity is a key factor particularly during brooding period. A minimum humidity level of 55% should be maintained in broiler houses. Ventilation may need to be adjusted to achieve desirable humidity level. At high altitudes, psychrometers and concomitant interpretive tables should be used to assure achieving target humidity level. Low humidity levels cause chicks to become dehydrated, which result in poor performance and mortality. At high altitude, a fogger system may need to be installed to achieve the target humidity. A fogger system is an evaporative cooling system that is mainly used to reduce heat stress. A fogger system consists of a high‐pressure pump, pipe line, fogger nozzles and timer unit. The system sprays the water in the form of very fine mist, which absorbs the heat and exit the house through exhausting fans. Managing the fogger system and ventilation system allows to reach the target humidity level.

### 7.4 Lighting programs

Many broiler breeders in high altitude areas use lighting programs to help reduce early body weights and hence ascites incidence. However, lighting programs are not recommended to apply until after 7 days of age in order to ensure proper heart and lung development in growing birds and to achieve 7‐day body weight targets. (Aviagens, 2009). Aviagen recommends a minimum 4 h darkness for broilers raised up to 2.5 kg staring from 8 days of age through slaughter. Several intermittent lighting schedules have been studied. Hassanzadeh et al. (2005) studied an intermittent lighting program consisting of 1L:3D cycles in comparison with a continuous lighting program (23D:1D) and they reported a significant reduction in ascites mortality. It should be noted that intermittent lighting programs can only be implemented in windowless houses.

## 8 BREEDING STRATEGIES AT HIGH ALTITUDE

Researchers at the University of Arkansas, Fayetteville, AR, developed three research lines from a commercial line through divergent selection for ascites resistance. The base population has been maintained under random mating without selection. Sib‐selection based on ascites phenotype of broilers kept in hypobaric chambers produced ascites resistant and ascites susceptible lines. Their study suggests a limited number of major genes controlling ascites in broilers. A series of genome‐wide association studies (GWAS) using single nucleotide polymorphism (SNP) panels identified a few candidate SNPs as associated with ascites phenotype. Recently, whole genome resequencing (WGR) identified 28 genomic regions where SNP clusters (100s– 1000s of SNPs) showed frequency bias with respect to ascites phenotype (Lee et al., 2022; Parveen et al., 2020). Two of these regions were validated by further genotyping of additional DNA samples and found to have potential epistatic interaction. One region spanned more than 120 kbp on chromosome 2 including the 3′ end of the gene for carboxypeptidase Q (CPQ). The second was an approximately 50 kbp region on chromosome 22 spanning the 3′ end of the gene for leucine‐rich repeat transmembrane neuronal 4 (LRRTM4).

Selection based on SNPs in CPQ and LRRTM4 gene regions resulted in a sex‐ and simulated altitude‐dependent reduction of ascites incidence in F2 cohorts of the marker‐assisted selected line with no significant negative impacts on zootechnical performance (Lee et al., 2022).

## 9 CONCLUSION

High altitude significantly impacts the growth performance and livability of broiler chickens. Commercial broiler strains fail to attain their genetic potential when rearing at highland regions. There are effective nutritional interventions and management practices that could be helpful to overcome the challenges of high altitude when raising broiler chickens. Although feed restrictions and medical drugs have shown to be effective in controlling ascites, these strategies have not gained attention. Manipulation of dietary composition along with an application of a wide range of nutraceuticals such as LC and guanidinoacetic acid and phytochemicals are more practicable and may be promising approaches to circumvent ascites occurrence in broilers reared at high altitude. Management strategies particularly during brooding period have been shown to be effective to overcome the situation. Strategy to breed specific broiler strains which are more suitable for high altitude regions would be recommended.

## CONFLICT OF INTEREST

The authors declare that there are no conflicts of interest.

## ETHICS STATEMENT

The Ethical Committee of Shahrekord University Research Council approved all procedures used in the study in accordance with the standard of 1964 Declaration of Helsinki.

### PEER REVIEW

The peer review history for this article is available at https://publons.com/publon/10.1002/vms3.784

## Data Availability

Date are ready upon request.
